# Calcaneus Metastasis from Follicular Thyroid Carcinoma 12 Years after Total Thyroidectomy

**DOI:** 10.1155/2018/5281452

**Published:** 2018-08-29

**Authors:** Mohammad Jawad H. Rahal, Karam M. Karam, Selim M. Nasser, Jihad A. Daher, Hicham G. Abdel Nour, Alexandre H. Nehme, Ramzi C. Moucharafieh

**Affiliations:** ^1^Department of Orthopedic Surgery and Traumatology, Clemenceau Medical Center Affiliated With Johns Hopkins International, Clemenceau, Beirut, Lebanon; ^2^Department of Orthopedic Surgery and Traumatology, Saint George University Medical Center, Balamand University, P.O. Box 166378, Achrafieh, Beirut 1100 2807, Lebanon

## Abstract

We report the case of a sixty-eight-year-old female patient who presented for left ankle pain; X-rays and MRI showed a benign calcaneal cyst, found to be delayed distant metastasis from primary papillary thyroid carcinoma. Patient required surgical excision of the cyst. Results of histological examination showed metastatic papillary thyroid carcinoma. The patient underwent total thyroidectomy 12 years prior to presentation, with the same pathology. Postoperatively, she was treated with radioactive iodine. At 10-year follow-up post calcaneal mass excision, she was found to have a right proximal tibial mass and found to be recurrent with the same pathology. This case reports a rare condition that will be taken into consideration in bone metastasis with thyroid cancer.

## 1. Introduction

Follicular thyroid carcinoma (FTC) accounts for 10–20% of all thyroid malignancies [1]. It is the second most common thyroid cancer [2]. Metastatic thyroid carcinoma to the bone was reported in the skull [3, 4] and the spinal column [5].

Malignant metastases to the calcaneus have been reported in cancer of the urinary tract system (prostate, bladder) [6, 7], the female genital system (uterine carcinosarcoma, endometrial carcinoma) [8, 9], and the lungs [10].

The aim of this paper is to report a very rare case in practice of delayed distant thyroid carcinoma metastasis to the calcaneus.

## 2. Case Report

This is a case of a 68-year-old female presented to the clinic for a 2-month history of recurrent left ankle pain, associated with swelling and edema. The patient had a history of thyroid follicular adenocarcinoma which was treated surgically with total thyroidectomy and postoperative iodine treatment 25 years prior to presentation. At 12 years post thyroidectomy, she was diagnosed with a calcaneal mass of the same pathology and diagnosed with delayed distant thyroid carcinoma metastasis to the calcaneus. 10 years after calcaneal mass excision, the patient was diagnosed with a proximal tibial mass that turned out to be also delayed metastasis of the same pathology.

At the first presentation 15 years ago, she initially presented complaining of mild edema of the left ankle with intermittent pain upon daily activity. Physical exam showed full range of motion of the ankle joint with intact motor power and intact neurovascular status. There were no cutaneous lesions.

**Figure 1:**
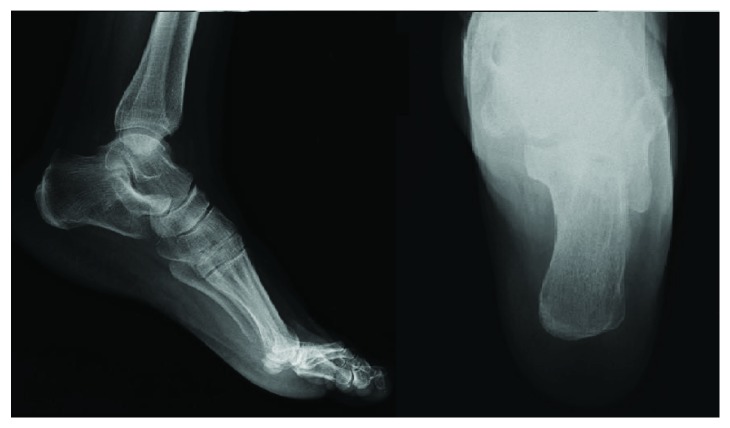
Retrospective assessment of the preoperative plain radiographs of the left foot shows a 3 cm oval lytic lesion in the anterior aspect of the calcaneum.

Plain radiographs of the left ankle showed a 3-centimeter oval lytic lesion in the anterior aspect of the calcaneum (Figure 1).

**Figure 2:**
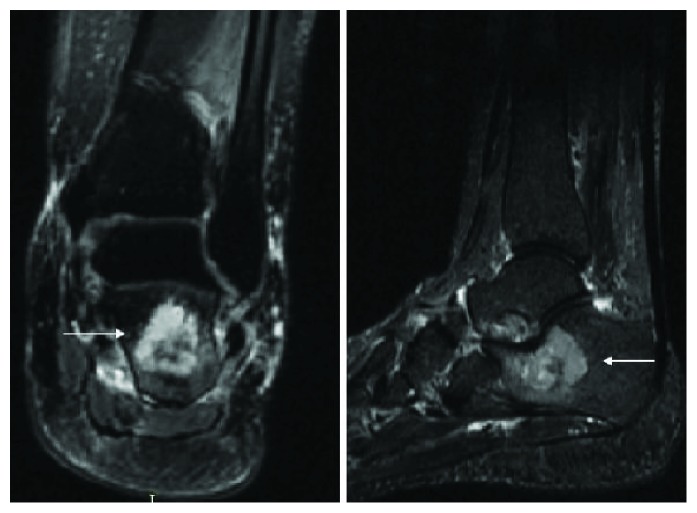
MRI of the left ankle: T2-WI with fat saturation sequences obtained in the coronal and sagittal planes confirm the presence of anterior calcaneal bone tumor (white arrows).

An MRI of the left ankle showed a 3.2 cm well-defined benign-looking lytic lesion of the calcaneal neck reaching the cortex which appeared to be mildly irregular with mild degenerative disease of the posterior subtalar joint consistent with an intraosseous ganglion cyst of the calcaneus (Figure 2).

**Figure 3:**
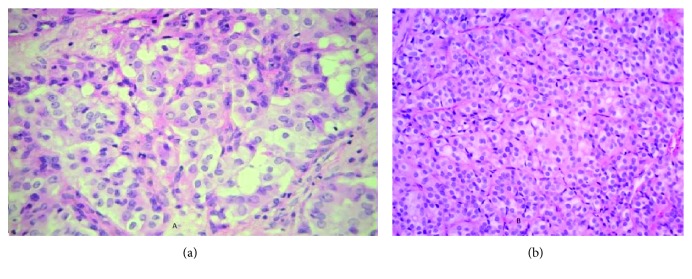
(a) Calcaneus: calcaneus lesion showing neoplastic thyroid follicular cells (H&E, ×200). (b) Thyroglobulin: neoplastic cells in the calcaneus lesion expressing thyroglobulin (thyroglobulin immunostains, ×200).

Medical treatment with NSAIDs and paracetamol was initiated, along with partial weight bearing and relative rest with no improvement at follow-up at 4 weeks. Surgical intervention was decided due to the persistent pain. The cyst was resected with a margin of surrounding fibro-osseous tissue and the bone grafted. Histopathological evaluation revealed a metastatic carcinoma of the thyroid gland. Immunostaining showed that the cells expressed cytokeratin, cytokeratin 7, and thyroglobulin, all of which confirm the diagnosis (Figure 3).

**Figure 4:**
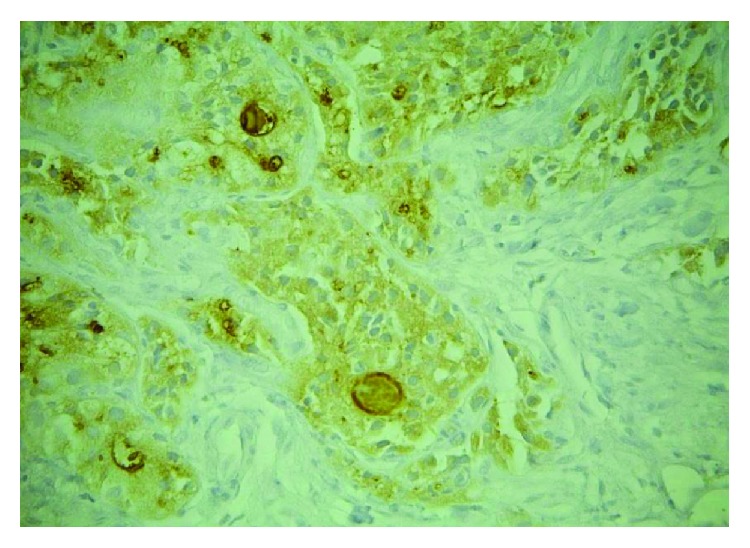
Thyroid: original follicular carcinoma in the thyroid (H&E, ×200).

For further confirmation, the recently excised cyst slides were compared to the pathology slides of the thyroid excision undertaken 12 years prior to the calcaneal presentation and were found out to be of the same pathology (Figure 4).

The patient had a smooth postoperative hospital stay and clinical recovery from pain before discharge; postoperative follow-up showed necrosis of the upper part of the wound which healed by secondary intention.

The patient had a complete bone metastasis workup; chest X-ray showed a right upper lobe nodule for which an FNA biopsy under CT scan was done demonstrating the same pathology as for the calcaneus. A bone scan was ordered showing no definitive sign suggesting metastasis with no specific abnormality of the manubrium-sterni joint and the right proximal metaphysis of the right tibia which was nonspecific for distant metastasis according to the nuclear radiologist. After the discussion with the oncologist, decision for radioactive iodine therapy was made.

**Figure 5:**
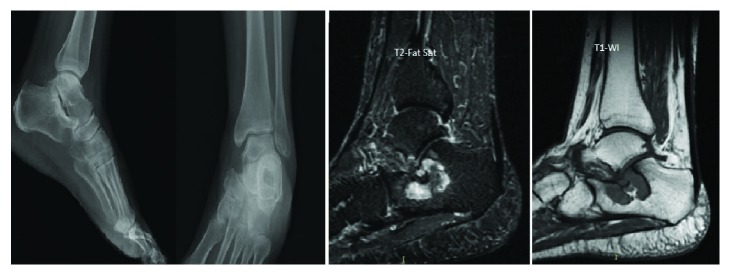
Postoperative plain radiographs and MRI (sagittal T1-WI and T2 fat sat sequences) reveal postsurgical changes in the calcaneus with decrease in the size of the mass which is partially replaced with marrow fat.

At 3-month postoperative follow-up, while the patient was undergoing chemotherapy, she recomplained of ankle pain upon ambulation, associated with edema. The patient had 5/5 motor strength, no numbness, and no sign of infection. A left ankle X-ray and MRI were ordered (Figure 5) to rule out any recurrence at the surgical site. The new investigations showed oval-shaped lytic lesion of 2.8 cm, and MRI showed increase in size of the calcaneal mass without pathologic fracture.

The patient was treated symptomatically; a follow-up MRI at 8 months showed postoperative enhancement in the surgical bed suggestive of viable tissue, and at 10 months, a follow-up MRI showed that there was near total healing of the calcaneal region.

**Figure 6:**
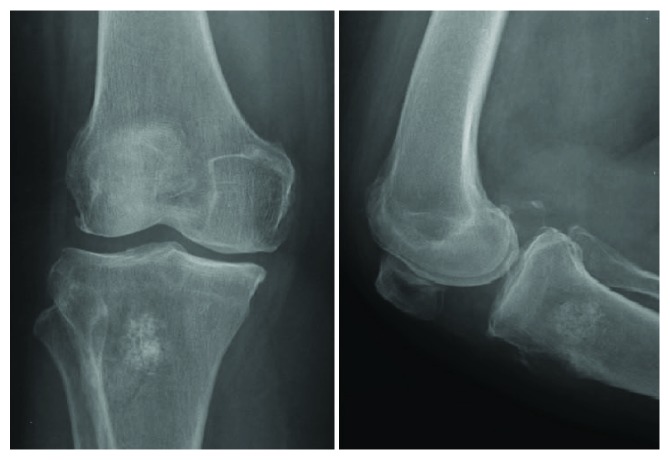
X-ray of the right knee reveals a mixed sclerotic and lytic lesion in the proximal aspect of the tibia with anterior cortical disruption.

**Figure 7:**
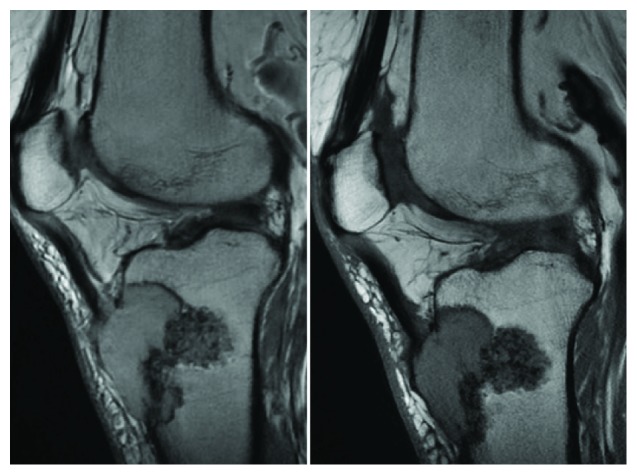
MRI of the right knee: sagittal T1-WI and T2-WI sequences of the knee show a 4.5 cm lytic expansile mass at the anterior proximal aspect of the tibia involving the patellar tendon insertion. A sclerotic area is seen along the posterior border.

After 10 years post calcaneal mass excision, keeping in mind that the patient was symptom-free with respect to her ankle since the surgery, the patient presented to the clinic complaining of recurrent right knee pain. Investigation showed right proximal tibial metastasis with invasion of the patellar tendon. Surgical resection of the metastasis with repair of the patellar tendon was performed, and pathology was also consistent with thyroid follicular cell carcinoma (Figures 6 and 7).

The oncologist was consulted, and the decision for treatment with teroglobulin as well as adjuvant radiation therapy was made.

## 3. Discussion

This is the first reported case of follicular thyroid carcinoma metastasis to the foot to our knowledge. Follicular thyroid carcinoma is slow growing and is associated with good prognosis. However, FTC has a poor prognosis in distant metastasis [11]. The lungs and bone are the most common sites of distant metastasis in FTC.

In addition, bone metastasis seems to be more aggressive than lung metastasis [12, 13]. The 10-year survival prognosis in bone metastasis from differentiated thyroid cancer is reported in the literature to be 27% [14]. The most distant metastasis reported case was to the skull [3, 15, 16] and vertebrae [17, 18].

We report a single late distant metastasis from thyroid cancer. The calcaneal involvement from this case was 12 years after the primary disease that was controlled through total thyroidectomy. Ogawa et al. described such a late presentation [4] 12 years after primary intervention but through thyroxine supplementation. Our patient underwent surgical excision of the calcaneal metastasis which is the treatment of choice, reported in the literature, improving the survival rate [19].

In contrast, Lin et al. report that surgical interventions directed at skeletal metastasis in FTC patients with overt multiple skeletal metastasis might not result in improvement of the overall survival [20].

Aggressive surgical treatment to remove metastatic lesions and postoperative I-131 therapy done to our case were reported as the recommended management and associated with the best long-term survival [21]. Follow-up was done for 10 years after the diagnosis of the distant metastasis.

The calcaneal involvement as single bone metastasis from primary tumor was reported in cases of primary prostate cancer where the treatment was radiotherapy [6]. It has also been reported in cases of superficial transitional cell carcinoma of the bladder even without muscle invasion [7]. The treatment of uterine carcinosarcoma, namely malignant mixed mullerian tumor with metastatic solitary bone metastasis to the calcaneus, consisted of wide surgical excision followed by adjuvant chemotherapy and radiotherapy [7]. In addition, isolated calcaneal metastasis has been seen in endometrial adenocarcinoma where the management was radiotherapy followed by below knee amputation [8]. Isolated calcaneal metastasis from lung carcinoma is a very rare entity reported with treatment by chemotherapy and local radiation [9].

## 4. Conclusion

This is a very rare and unusual case of calcaneal distant metastasis from follicular thyroid carcinoma 12 years after tumor excision by total thyroidectomy and iodine treatment. The patient underwent surgical excision of the calcaneal tumor and bone grafting followed by I-131 therapy. The 10-year follow-up post diagnosing calcaneal metastasis revealed another delayed recurrence at the proximal tibia of the contralateral limb.

It is recommended that follicular cell carcinoma should be considered in the differential diagnosis of bone cyst in any patient with history of FTC even years after primary therapy.
